# Analysing the determinants of healthcare insurance uptake in Nigeria

**DOI:** 10.1186/s12913-025-13422-0

**Published:** 2025-10-06

**Authors:** Wasiu Adekunle, Oluwaseyi Vincent

**Affiliations:** Research and Development Department, The Nigerian Economic Summit Group (NESG), Lagos, Nigeria

**Keywords:** Healthcare, Insurance uptake, Socioeconomic factors, NHIS, NHIA, C35, I13, O15

## Abstract

Low health insurance coverage has remained a persistent challenge in Nigeria, restricting access to healthcare despite the coexistence of various insurance schemes. While previous research has primarily focused on the National Health Insurance Scheme (NHIS) or community-based health insurance (CBHI), few studies have collectively examined the factors influencing enrolment across different types of insurance—namely, community-based, employer-sponsored, and private schemes. This study addresses this research gap using household data from the 2018 Nigeria Demographic and Health Survey (DHS). Employing logit and probit regression models, the analysis explores the likelihood of health insurance enrolment. The results reveal that six socioeconomic factors—education attainment, age of the household head, bank account ownership, wealth status, marital status, and employment type—significantly influence enrolment in both overall insurance (all types of insurance) and employer-sponsored insurance. However, these factors vary in importance for community-based and private insurance. For instance, individuals with low education have a higher likelihood of enrolment in CBHI than those with high education. The study also finds that financial constraints may reduce the possibility of enrolment among low-income groups compared to their middle- and high-income counterparts. The findings underscore the need for targeted interventions to enhance enrolment, particularly among individuals with lower educational levels, limited income, and those employed in the informal sector.

**Supplementary Information:**

The online version contains supplementary material available at 10.1186/s12913-025-13422-0.

## Introduction

Health insurance provides an easy avenue to access healthcare and is intended to serve as relief from the out-of-pocket (OOP) spending burden, easing financial constraints. However, a recent estimate suggests that access to formal health insurance in Nigeria is 5–7% of the total population [1]. This low coverage rate could be due to limited government spending on healthcare. For instance, in the 2025 approved budget, the Nigerian government allocated N2.38 trillion to the health sector, accounting for 4.33% of the total budget, well below the 15% commitment set by the 2001 Abuja Declaration [2]. Consequently, Nigerians have resorted to out-of-pocket payments, accounting for 75% of total current health spending in the country [3], thereby heightening poverty risks and raising concerns about Nigeria’s ability to achieve Sustainable Development Goal (SDG) target 3.8 [see 4, 5, 6].

In recent years, the Nigerian government has made efforts to improve healthcare access, with notable instances including the establishment of the National Health Insurance Scheme (NHIS) in 1999, but it was not operational until 2005. The scheme was made mandatory only for formal workers in both the public and private sectors. By implication, after almost two decades of inception, the NHIS could only cover about 5% of Nigerians [7]. Aside from the supply-side failure of the scheme, low enrolment has also hindered its success [8]. The poor enrolment could be attributed to the large informal sector, which employs over 90% of the workforce [9, 10]. The supply-side constraints include an inadequate legal framework, an optional enrolment policy, poor funding, and a lack of political will [11].

To salvage the National Health Insurance Scheme (NHIS) from the brink of collapse, the Federal Government of Nigeria established the National Health Insurance Authority (NHIA) Act in 2022, which made health insurance mandatory for all citizens and legal residents [12]. However, using compulsion alone may not guarantee the expected outcomes; hence, there is a need to uncover the factors underlying the low health insurance uptake in Nigeria. Corruption allegations, accusations of misappropriation of funds, and poor quality of healthcare delivery have been reported as disincentives to enrolment in health insurance [12–15]. Meanwhile, these are supply-side factors. Understanding the sources of low health insurance uptake from a demand-side perspective could inform strategies to enhance coverage. This study fills this gap. The existing empirical literature on health insurance has generally relied on primary data collection through interviews and questionnaires, which may not accurately represent the Nigerian population [see 16, 17, 18]. Also, past Nigerian-based studies have primarily focused on NHIS and the Community-based insurance [see 19, 14, 5, 17], while ignoring other forms of insurance, particularly employer-sponsored and private health insurance schemes, largely considered in non-Nigerian-based studies [see 20, 21].

Moreover, the current study examines the determinants of health insurance uptake using Nigeria’s most recent Demographic and Health Survey (DHS) data covering 36 states and the Federal Capital Territory (FCT). By disaggregating uptake into employer-sponsored, community-based, and private insurance, our study provides a more detailed understanding of how different determinants interact with scheme design and target populations. For example, while wealth and type of employment have a more substantial influence on employer-sponsored insurance enrolment, they have weaker or inconsistent effects on community-based schemes. This distinction is crucial for policymakers seeking to expand coverage, as it suggests that strategies must be tailored not only to population characteristics but also to the specific features of each insurance model.

Following the introduction, this paper is rendered in four sections as follows. Section Two contains the Literature Review. Section Three entails the Methodology. Section Four presents the Results and Discussion, while Section Five concludes the study.

## Literature review

### Theories of demand for health insurance

Theories explaining the demand for health insurance are grounded in economic theories of consumer behaviour and decision-making under uncertainty. This review focuses on foundational frameworks, such as Expected Utility Theory (EUT), Cumulative Prospect Theory (CPT), and State-Dependent Utility Theory, as well as extensions that explicitly address the behaviour of low-income individuals. These extensions include theories that incorporate liquidity constraints, time preferences, and trust in informal insurance schemes. These theoretical perspectives are relevant to understanding the differentiated patterns of enrolment identified in our study.

*Expected Utility Theory (EUT)* remains a central model for explaining the uptake of health insurance under uncertainty. While it is theoretically applicable across income groups, its assumptions—such as risk aversion and the ability to absorb certain losses—align more closely with the behaviour of formally employed or higher-income individuals, who often have more financial stability and capacity to plan for future risks. The theory assumes that individuals are risk-averse and make decisions to maximise their expected utility when faced with uncertainty [22]. Health insurance is viewed as a mechanism to avoid the uncertain financial loss associated with illness, even at the cost of a certain premium. According to EUT, risk-averse individuals will choose to pay a known premium to avoid the possibility of catastrophic health expenditures. However, EUT does not sufficiently explain the behaviour of low-income individuals, who, despite being more vulnerable to health shocks, are less likely to enroll.

This limitation has led to the emergence of alternative theories, such as *Cumulative Prospect Theory (CPT)*, which provides a more nuanced behavioural explanation. CPT suggests that individuals do not evaluate outcomes based on final utility levels but rather as gains or losses relative to a reference point [23]. People tend to overweight small probabilities and underweight large ones and are more sensitive to losses than to gains [24, 25]. In the context of health insurance, this means that individuals may avoid paying premiums because the immediate financial loss feels more salient than the uncertain benefit of protection against future illness.

*State-Dependent Utility Theory* further refines this perspective by arguing that individuals’ preferences and risk aversion are influenced by their current state, such as age, health, or economic status [25], As people age or experience declining health, they are more likely to seek health insurance to mitigate future risks. This theory also helps explain why wealthier individuals may exhibit greater risk aversion and a higher likelihood of enrolment, given their greater financial capacity and health awareness.

Another relevant perspective is the *theory of insurance demand among the poor*, which challenges the applicability of traditional economic models, such as the Expected Utility Theory (EUT), to low-income populations [25] , and related literature highlight how liquidity constraints, limited access to financial tools, and short-term time preferences shape the behaviour of poor households. These constraints often lead to risk-seeking behaviour in the face of loss and an aversion to investing in uncertain or delayed benefits, such as health insurance. The poor may not only be unable to afford premiums but may also prioritise immediate consumption needs over uncertain future protection.

Taken together, these theories offer a robust framework for understanding the determinants of health insurance uptake revealed in our study. While the EUT remains relevant for explaining enrolment among formal workers and wealthier groups, behavioural models like CPT and state-dependent utility provide deeper insights into the under-enrolment of vulnerable populations. The theory of insurance demand among the poor adds further layers of complexity, particularly when examining CBHI and informal sector dynamics. By disaggregating health insurance into employer-sponsored, community-based, and private types, our study is better positioned to align theoretical insights with empirical realities. This approach enables more targeted policy recommendations, acknowledging that the motivations and barriers to enrolment vary significantly across population segments and insurance types.

### The empirical literature

In this sub-section, the study reviews existing empirical evidence on the drivers of health insurance enrolment. Health insurance uptake in Nigeria has been the subject of extensive scholarly inquiry, particularly in relation to the socioeconomic and demographic characteristics that influence enrolment. Numerous studies have consistently shown that variables such as education, income level/wealth status, employment status, age of household head, and marital status play a significant role in determining whether individuals choose to enroll in health insurance schemes [5, 14, 26–29]. While this body of work offers valuable insights, a limitation of many previous studies is the tendency to treat health insurance as a homogenous category. The current study contributes to the literature by distinguishing the different types of health insurance, thereby revealing the differentiated role that various socioeconomic and demographic factors play across these distinct schemes.

Educational attainment has long been identified as a strong predictor of health insurance uptake. Individuals with higher levels of education are more likely to be aware of available schemes, understand their benefits, and navigate enrolment procedures. [5], using 2013 DHS data, found that women with secondary or tertiary education were significantly more likely to enroll in the NHIS. Similarly, [14] reported that higher education levels were associated with greater insurance coverage in both Nigeria and South Africa.

Employment status is another crucial factor influencing insurance uptake. Studies, such as those by [30], have found a positive relationship between formal employment and health insurance enrolment. Similarly, [31] identified employment type as a significant predictor of participation in health insurance schemes in Zimbabwe. Marital status has also been shown to affect health insurance enrolment. Married persons often have greater healthcare needs and are more risk-averse, making them more likely to seek financial protection through insurance. [29, 32] found positive associations between marital status and insurance uptake.

Evidence from Nigeria and other sub-Saharan African countries indicates that the age of the household head is positively associated with health insurance uptake. The study by [28] showed that households headed by older individuals are more likely to be enrolled in insurance schemes, particularly due to a greater perceived vulnerability to health shocks. Also, an emerging but underexplored factor in the literature is financial inclusion, particularly access to banking services. While not widely studied in Nigeria, there is growing international evidence that bank account ownership facilitates insurance uptake by simplifying premium payments and reducing transaction costs. The study by [33] indicated that health insurance improves welfare in Nigeria. Hence, individuals with greater health care needs will demand more health care services with health insurance, aligning with the Anderson Model.

Moreover, several studies have also showcased the socioeconomic factors that influence the uptake of specific health insurance types, following the Andersen model (which distinguishes between predisposing, enabling and needs-based factors). For instance, utilising the logistic regression, [34] established that the significant factors influencing the decision to purchase private health insurance (PHI) in China are age, education, marital status, household size (known as predisposing factors), household income, social basic medical insurance status, geographical factors, household medical expense, and medical debt (called enabling factors), and needs-based factors (health status). Similarly, [35] have also identified employment status, household income and location of residence as significant drivers for PHI uptake in East Coast Malaysia. Meanwhile, families with lower incomes, those with lower parental education, families where parents work in smaller establishments, and families in which neither parent has union representation are all less likely to have access to employer-sponsored health insurance. [36] [37] demonstrated that financial inclusion and employment level are positive drivers of private health insurance uptake across Nigerian states.

In addition, leveraging logistic regression analysis, [21] revealed that family size, history of illness within the household, perceived amount of membership contribution, being married, and trust in the programme were determinants of increased enrolment decisions in CBHI in Northwest Ethiopia. Meanwhile, [20] demonstrated that factors negatively affecting CBHI membership are old age, low education, low household income, poor quality of care, lack of trust in providers, remoteness, rules considered too strict or inappropriate, low trust in administrators, and inadequate information campaigns.

In summary, these findings emphasise the need for policy approaches tailored to the diverse barriers and incentives across different health insurance schemes. However, most Nigerian studies have focused narrowly on the National Health Insurance Scheme (NHIS) or community-based health insurance (CBHI), without systematically examining the broader array of insurance types that citizens access. This study fills that gap by leveraging the 2018 Nigeria Demographic and Health Survey [38], which disaggregates coverage into employer-sponsored, community-based, private/commercially purchased, and social security insurance. By analysing these schemes separately, the study provides a nuanced understanding of how socioeconomic factors influence enrolment across different insurance models and target groups. This study also advances prior work by employing logit and probit models for robustness, similar to some studies [see 39, 4].

## Methodology

### Model specification

This study adopts the theory of demand for health insurance among the poor. While the expected utility theory makes assertions about individual behaviour under uncertainty, it makes no assertions about the effect of socioeconomic status on the demand for healthcare insurance. The theory of demand for health insurance among the poor ultimately fills this gap [25]. We also draw insights from Andersen’s behavioural model, developed by Ronald Andersen in 1968, which indicates three factors influencing healthcare service utilisation to include predisposing, enabling and evaluated need factors [40].

First, the predisposing factors include demographic structures (age, marital status and gender), social structures (education, occupation, social networks, culture and ethnicity), and health beliefs (knowledge and perception about health and health services). Second, the enabling factors include resource availability (health personnel and facilities), and means and know-how to get to those services and make use of them (income, health insurance, a regular source of care, and travel and waiting time). Lastly, the evaluated need factors represent professional judgment about people’s health status and their need for medical care due to illnesses, diseases, and physical conditions [41].

In line with this theory of demand for health insurance among the poor, Andersen’s behavioural model, and the extant empirical literature, the current study employs six (6) critical socioeconomic factors that could influence healthcare insurance uptake in Nigeria. These include educational level, age of the household head, having a bank account, wealth level, marital status, and employment type. However, we included a variable known as financial inclusion (measured as having a bank account), which is not explicitly stated in the Andersen model, but which we can situate within the enabling factors. This is because financial inclusion and insurance could work complementarily [42].

Therefore, we specify four separate models for all types of insurance (overall insurance), community-based insurance, employer-sponsored insurance, and private insurance, respectively, as follows:

1 $$\begin{aligned}\:{insured}_{i}=&{\beta\:}_{0}+{\beta\:}_{1}{educ\_level}_{i}+{\beta\:}_{2}{age\_hh}_{i}\\&+{\beta\:}_{3}{own\_bank\_acc}_{i}+{\beta\:}_{4}{wealth}_{i}\\&+{\beta\:}_{5}{marital}_{i}+{\beta\:}_{6}{employ\_type}_{i}+{\epsilon\:}_{i}\end{aligned}$$

2 $$\begin{aligned}\:{insure\_cbi}_{i}=&{\beta\:}_{0}+{\beta\:}_{1}{educ\_level}_{i}+{\beta\:}_{2}{age\_hh}_{i}\\&+{\beta\:}_{3}{own\_bank\_acc}_{i}+{\beta\:}_{4}{wealth}_{i}\\&+{\beta\:}_{5}{marital}_{i}+{\beta\:}_{6}{employ\_type}_{i}+{\epsilon\:}_{i}\end{aligned}$$

3 $$\begin{aligned}\:{insure\_ebi}_{i}=&{\beta\:}_{0}+{\beta\:}_{1}{educ\_level}_{i}+{\beta\:}_{2}{age\_hh}_{i}\\&+{\beta\:}_{3}{own\_bank\_acc}_{i}+{\beta\:}_{4}{wealth}_{i}\\&+{\beta\:}_{5}{marital}_{i}+{\beta\:}_{6}{employ\_type}_{i}+{\epsilon\:}_{i}\end{aligned}$$

4 $$\begin{aligned}\:{insure\_pri}_{i}=&{\beta\:}_{0}+{\beta\:}_{1}{educ\_level}_{i}+{\beta\:}_{2}{age\_hh}_{i}\\&+{\beta\:}_{3}{own\_bank\_acc}_{i}+{\beta\:}_{4}{wealth}_{i}\\&+{\beta\:}_{5}{marital}_{i}+{\beta\:}_{6}{employ\_type}_{i}+{\epsilon\:}_{i}\end{aligned}$$

All the variables stated in Eqs. 1–4 are described in Table 1. Since the dependent variable is a binary response variable (yes/no), which in this case is whether an individual has health insurance or does not, we employ the widely used qualitative response models, including Logit and Probit regression. Both models are non-linear probability models that link predictors to the probability of an outcome, but differ in terms of the error term distribution assumption: Logit follows a logistic distribution while Probit follows a standard normal distribution. Since the logistic and normal cumulative distribution functions (CDFs) are very similar (both are symmetric and S-shaped), the two models often produce qualitatively similar results. Using both provides a robustness test: if the results are consistent across logit and probit, then your findings are not dependent on distributional assumptions. For instance, marginal effects, significance levels, and direction of relationships should not change drastically between the two [43]. It is for this reason that we explore both models.

**Table 1:** Description of variables

Symbol	Definition and Unit of Measurement
$\:{insured}_{i}$	Equals 1 if an individual is enrolled for any type of health insurance, and 0 if an individual does not have any health insurance.
$\:{insure\_cbi}_{i}$	Equals 1 if an individual is enrolled for community-based health insurance (CBHI), and 0 if an individual does not have CBHI.
$\:{insure\_ebi}_{i}$	Equals 1 if an individual is enrolled for employer-sponsored health insurance, and 0 if an individual does not have employer-sponsored health insurance.
$\:{insure\_pri}_{i}$	Equals 1 if an individual is enrolled for private/commercially purchased health insurance, and 0 if an individual does not have private health insurance.
$\:{educ\_level}_{i}$	Equals 1 if an individual has secondary and tertiary education (classified as the high education group), and 0 if an individual has primary and no education (classified as the low education group).
$\:{age\_hh}_{i}$	Age of household head (years).
$\:{own\_bank\_acc}_{i}$	Equals 1 if an individual has a bank account and 0 if an individual does not have a bank account.
$\:{wealth}_{i}$	This implies wealth level and is equal to 1 if an individual belongs to a high-income or middle-income group, and 0 if an individual belongs to a low-income group. The Nigeria Demographic and Health Survey (DHS) divides households into five wealth quintiles: poorest, poorer, middle, richer, and richest. For this study, the poorest and poorer are classified as the low-income group; the middle quintile as the middle-income group; the richer and richest as the high-income group.
$\:{marital}_{i}$	Equals 1 if an individual is married, and 0 if an individual is single or unmarried.
$\:{employ\_type}_{i}$	Equals 1 for year-round employment, and 0 if an individual is engaged in seasonal/occasional employment.

### The logit regression

The Logit regression follows a logistic distribution. The current case indicates that the probability that a household member is covered by health insurance ($\:{P}_{i}$) is a non-linear function of the explanatory variables ($\:{X}_{i}^{{\prime\:}}s$), including educational level, age of the household head, having a bank account, wealth level, marital status, and employment type.

5 $$\:{P}_{i}=E\left({Y}_{i}=1|{X}_{i}\right)=\frac{1}{1+{e}^{{-Z}_{i}}}=\frac{{e}^{z}}{1+{e}^{{z}_{i}}}$$

Where 

6 $$\:{Z}_{i}={\beta\:}_{0}+{\beta\:}_{1}{X}_{i}$$

Equation (6) is called the (cumulative) logistic distribution function. The use of the logit model is justified on the following grounds. First, it helps to overcome the limitations of the Linear Probability Model (LPM), which often predicts probabilities outside the [0,1] range. The logit model, by using the logistic cumulative distribution function (CDF), ensures predicted probabilities are always between 0 and 1 [44]. Second, a major strength of logit is that coefficients can be expressed as odds ratios, which are intuitive for explaining the likelihood of events [45]. Lastly, the logit framework easily extends to multinomial logit (for outcomes with more than two categories) and conditional logit (for choice models), thereby making its application more versatile [46].

The probability that a household does not fall below the poverty line can be derived as:

7 $$\:1-{P}_{i}=\frac{1}{1+{e}^{{z}_{i}}}$$

Next, the odds ratio is obtained by dividing the probability of success ($\:{P}_{i})\:$by the probability of failure (1-$\:{P}_{i}$), that is,

8 $$\:\frac{{P}_{i}}{{1-P}_{i}}=\frac{1+{e}^{{z}_{i}}}{1+{e}^{{-Z}_{i}}}={e}^{{z}_{i}}$$

Taking the natural log of the odds ratio gives

9 $$\:{L}_{i}=\text{ln}\left(\frac{{P}_{i}}{{1-P}_{i}}\right)={Z}_{i}={\beta\:}_{0}+{\beta\:}_{1}{X}_{i}$$

Where $\:{L}_{i}$ is referred to as the logit and is expressed as a linear function of the covariates $\:{X}^{{\prime\:}}s$, as defined previously.

### The probit regression

The probit regression is employed to generate a robust analysis. In the probit approach, the inverse standard normal distribution of the probability is modelled as a linear combination of predictors. For example, suppose a response variable $\:Y$ is binary, meaning it can have only two possible outcomes, which we will denote as 1 and 0. In this case, the response variable is whether a household member is covered by health insurance or not. There is also a vector of explanatory variables $\:X$, which is assumed to influence the outcome Y. Specifically, we assume that the model takes the form:

10 $$\:{Pr}\left(Y=1|X\right)=\varnothing\:\left({X}^{T}\beta\:\right)$$

where $\:Pr\:$denotes probability and $\:\varnothing\:$ is the cumulative distribution function of the standard normal distribution. The $\:\beta\:$ is the parameter estimate. It is possible to motivate the probit model as a latent variable model.

Suppose there exists an auxiliary random variable:

11 $$\:{Y}^{*}={X}^{T}\beta\:+\epsilon\:$$

where

12 $$Y\;=\;\left\{\begin{array}{l}=1\;if\;Y\ast,i.e.-\varepsilon\;<\;X^T\;\beta,\\=0\;if\;other\;wise\end{array}\right.$$

 

Then, Y can be viewed as an indicator of whether this latent variable is positive:

13 $$\:Y=\{\begin{array}{c}\:\:\:\:\:\:\:\:\:\:=1\:if\:{Y}^{*},\:i.e.\:-\epsilon\:<{X}^{T}\beta\:\\\:=0\:if\:otherwise\end{array},$$

where the response variable $\:Y$ is the likelihood of having health insurance coverage, and covariates $\:X$ are the possible socioeconomic determinants of health insurance uptake among Nigerians.

### Expected results

Health insurance uptake is expected to be positively related to gainful employment, particularly in the case of employer-sponsored and privately purchased insurance plans. Financial inclusion—such as having access to a bank account—may also facilitate premium payments and increase the likelihood of enrolment. Similarly, individuals with a stable source of income are more likely to seek quality healthcare, whether through direct out-of-pocket payments or by enrolling in a health insurance scheme. However, the uptake of health insurance could differ across different income groups, with low-income groups having low uptake of health insurance, while middle- and high-income groups have higher uptake. It is expected that married individuals are more likely to seek health insurance to mitigate financial risks associated with family healthcare responsibilities. Furthermore, higher educational attainment is expected to enhance awareness and understanding of the benefits of health insurance, thereby increasing enrolment rates. Finally, the age of the household head is likely to be positively associated with uptake, as older individuals may anticipate greater healthcare needs and seek financial protection, especially during retirement.

### Data source and Estimation technique

The data for all variables employed in this study were obtained from the 2018 Nigeria Demographic and Health Survey (DHS) (see Table 1 for variable description). The survey was implemented by the National Population Commission (NPC) in collaboration with the National Malaria Elimination Programme (NMEP) of the Federal Ministry of Health. Funding support came from the United States Agency for International Development (USAID), the Global Fund, the Bill and Melinda Gates Foundation (BMGF), the United Nations Population Fund (UNFPA), and the World Health Organisation (WHO). Technical assistance was provided by ICF International through the DHS Programme, a USAID-funded project that supports the design and implementation of population and health surveys worldwide.

The 2018 Nigeria DHS is a nationally representative survey covering all 36 states and the Federal Capital Territory. It sampled about 42,000 households, yielding interviews with 42,121 women aged 15–49 and 13,311 men aged 15–59. After data cleaning, 48,262 respondents were retained, representing nearly 87% of the total eligible sample (55,432 individuals). The survey’s primary objective was to generate up-to-date estimates of demographic and health indicators—including fertility, family planning, maternal and child health, nutrition, childhood mortality, malaria, HIV/AIDS awareness, and health insurance coverage—using standardised DHS instruments that ensure cross-country comparability [38].

In this study, both logit and probit models were estimated using the Maximum Likelihood Estimator (MLE) rather than Ordinary Least Squares (OLS). Binary outcomes violate key OLS assumptions, producing heteroscedastic errors and predicted probabilities outside the logical [0,1] interval. By contrast, MLE yields estimates that are consistent, asymptotically efficient, and normally distributed [44, 47, 48]. Additionally, the DHS covers four specific types of health insurance, namely, community-based, employer-sponsored, private and social security health insurance. However, since a handful of respondents are enrolled in more than one type of health insurance (refer to **Fig. 3**), we have removed these respondents from our analysis and focused on those with a single type of health insurance for precision. We have also excluded respondents enrolled in social security insurance from our analysis because there were fewer respondents associated with it. This is because regression based on small sample sizes may present biased and unreliable results, considering that MLE is well suited for large samples.

## Results and discussion

### Descriptive analysis of health insurance and socioeconomic characteristics

**Fig. 1:**
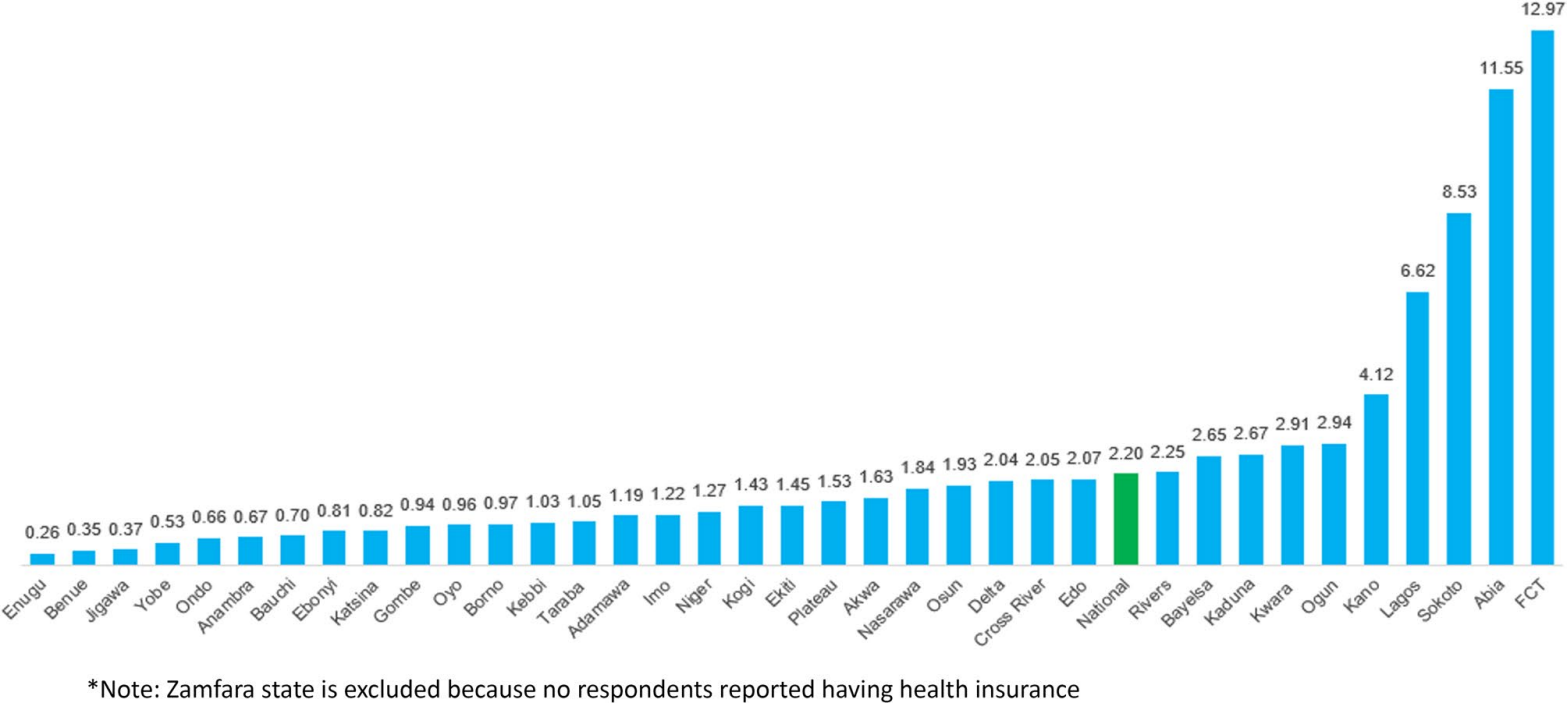
Health insurance coverage by states (%)*. *note: Zamfara state is excluded because no respondents reported having health insurance. source: Authors’ computation from Nigeria DHS 2018

Health insurance coverage in Nigeria is limited, but it is disproportionately concentrated in the country’s major economic and business hubs. The rate of health insurance coverage used in this study is the proportion of respondents (Nigerian residents) enrolled in health insurance. Using the 2018 Nigeria DHS data, the national average health insurance coverage rate as of 2018 was 2.2%. This indicates that approximately 2 out of 100 Nigerians are enrolled in health insurance (see Fig. 1). Nine (9) states and the FCT have coverage rates that are above the national average, with FCT recording the highest rate of 12.97%, followed by Abia (11.55%), Sokoto (8.53%), Lagos (6.62%), and Kano (4.12%). This top 10 list has the country’s major economic and business hubs. Being the seat of the Federal Government, Abuja hosts the federal government ministries, parastatals, and agencies, which are the largest employers of formal sector workers in Nigeria. Similarly, Abuja has an increased presence of consulates, embassies, high commissions, and country offices of many multinational and multilateral organisations.

**Fig. 2:**
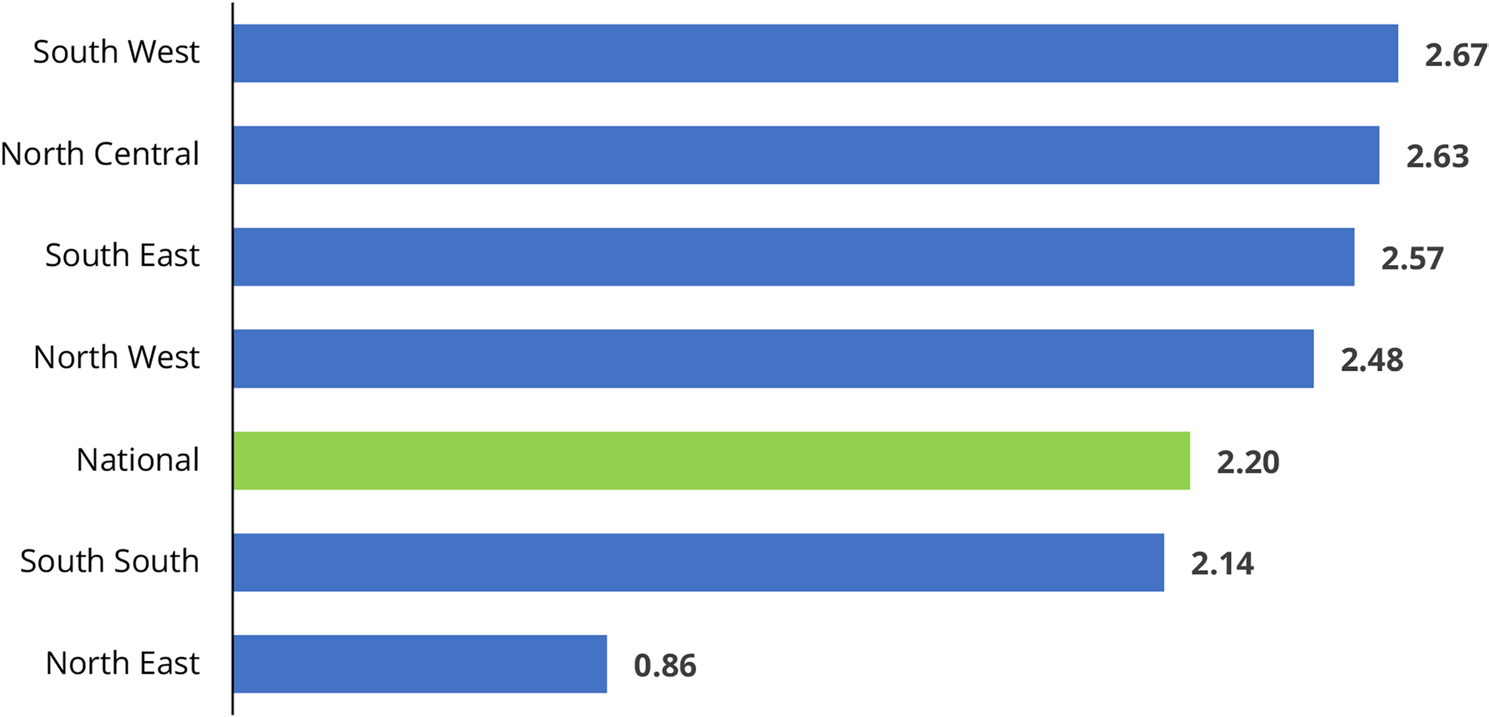
Health insurance coverage by region (%). source: Authors’ computation from Nigeria DHS 2018

Southern Nigeria has more comprehensive health insurance coverage compared to the Northern region. Regionally, the South West has the highest rate of health insurance coverage, at 2.67%, whereas the North East has the lowest rate, at 0.86% (see Fig. 2). On average, the Southern region (2.46%) outperforms the Northern region (1.99%). This disparity could be attributed to cultural and socioeconomic barriers, which are more prevalent in the Northern region than in Southern Nigeria. Therefore, to ensure a balanced enrolment across the six geopolitical zones in the country, there is a need to address the underlying cultural and socioeconomic impediments. This is the focus area of this study.

Employer-sponsored insurance is the most prominent type of health insurance in Nigeria. Approximately 70% of total respondents with health insurance (1,060) are enrolled directly by their employers (see Table 2). This is followed by those with community-based insurance (18.7%) and private insurance (8.5%). Social Security has the lowest count of enrollees (1.5%)^1^. Additionally, 1.8% of the respondents are enrolled in more than one health insurance. Having more than one health insurance helps enrollees reduce delays in accessing care, particularly in health centres that may favour one type of health insurance over another.

**Table 2:** Distribution of health insurance types

Type of Health Insurance	No. of Respondents	% Distribution
Employer-sponsored only	737	69.5
Community-based only	198	18.7
Private/commercially purchased only	90	8.5
Social Security only	16	1.5
Community-based & Private	8	0.8
Employer-sponsored & Private	6	0.6
Social Security & Private	4	0.4
All types of Health Insurance	**1**,**060**	**100.0**

Source: Authors’ Computation from Nigeria DHS 2018

The distribution of respondents across socioeconomic characteristics reveals striking imbalances. The proportion of respondents without bank accounts is four times greater than that of account holders (see Table 3). Educational level follows a similar pattern of disparity, with more than half of respondents having little or no schooling. Household and economic profiles reinforce these divides. The average age of household head reflects the predominance of the workforce (15–64 years), accounting for over 90% of the total sample size, suggesting that the majority of the respondents are gainfully employed. Low-income respondents nearly double the size of the middle-income group, with a smaller yet notable share in the high-income class. Socially, the sample is overwhelmingly married, with singles forming only a small fraction. Employment patterns also tilt heavily towards year-round work, with full-time engagement nearly tripling the number of seasonal or occasional workers.

**Table 3:** Description of socioeconomic characteristics

Socioeconomic Characteristics		Number of Respondents with Health Insurance	% Distribution
Own Bank Account	Yes	8,900	18.44
	No	39,362	81.56
Level of Education	No education	18,792	38.94
	Primary education	8,326	17.25
	Secondary education	16,860	34.93
	Tertiary education	4,284	8.88
Average Age of Household Head	Children (0–14 years)	4	0.38
	Youth (15–24 years)	1,030	2.13
	Workforce (15–64 years)	44,408	92.01
	Aged (65 years and above)	2,820	5.48
Income Class	Low-income	20,748	42.99
	Middle-income	10,712	22.20
	High-income	16,802	34.81
Marital Status	Married	44,090	91.36
	Not married	4,172	8.64
Employment Type	Occasional/Seasonal	12,906	26.74
	Year-round	35,356	73.26
Total respondents		48,262	100.00

Source: Authors’ Computation from Nigeria DHS 2018

The cross-tabulation between selected socioeconomic characteristics and enrolment for any type of health insurance yields varying results. First, more than two-thirds of enrolled respondents have bank accounts, implying that being financially included could complement enrolment in health insurance (see Table 4). Second, respondents with tertiary education have the highest enrolment level, followed by those with secondary education. This suggests a positive correlation between education level and health insurance uptake. Third, the respondents belonging to the workforce age range (15–64 years) have the strongest correlation with enrolment in at least one type of health insurance. Fourth, high-income households have the greatest enrolment level, while low-income households have the least enrolment level (see Table 4). This suggests a positive correlation between wealth level and health insurance uptake. Fifth, enrolment is more prevalent among married respondents. Finally, year-round employment is highly correlated with enrolment in health insurance compared to occasional or seasonal employment.

**Table 4:** Health insurance uptake and key socioeconomic characteristics

Socioeconomic Characteristics		No. of Respondents with all Health Insurance types	% of the Total Respondents with all types of Health Insurance
Own Bank Account	Yes	716	67.55
	No	344	32.45
Level of Education	No education	126	11.89
	Primary education	32	3.02
	Secondary education	284	26.79
	Tertiary education	618	58.30
Average Age of Household Head	Youth (15–24 years)	4	0.38
	Workforce (15–64 years)	1,028	96.98
	Aged (65 years and above)	28	3.24
Income Class	Low-income	94	8.87
	Middle-income	82	7.74
	High-income	884	83.39
Marital Status	Married	1020	96.23
	Not married	40	3.77
Employment Type	Occasional/Seasonal	116	10.94
	Year-round	944	89.06
Total respondents with health insurance		1060	100

Source: Authors’ Computation from Nigeria DHS 2018

### Factors determining health insurance enrolment in Nigeria

Tables 5 and 6 present the average marginal effects associated with the logit and probit regression models of health insurance uptake, respectively (see the Supplementary Material for the log of odds estimates for the logit model and z-score estimates for the probit model). As noted earlier, there will be four variants of the logit and probit models: overall health insurance, community-based insurance, employer-sponsored insurance, and private insurance.

**Table 5:** Logit regression results (average marginal effects)

VARIABLES	$\:insured$	$\:insure\_cbi$	$\:insure\_ebi$	$\:insure\_pri$
$\:educ\_level$	0.019***	−0.0019***	0.0263***	0.0027***
	(0.0022)	(0.00065)	(0.00296)	(0.00083)
$\:age\_hh$	0.00014***	0.000037*	0.0001***	0.000014
	(0.00054)	(0.00002)	(0.000036)	(0.00001)
$\:own\_bank\_acc$	0.031***	0.0032***	0.025***	0.0023***
	(0.0018)	(0.00075)	(0.0016)	(0.00051)
$\:wealth$	0.017***	0.00052	0.0312***	0.0051***
	(0.0026)	(0.00068)	(0.0051)	(0.0018)
$\:marital$	0.021***	0.0022	0.01978***	0.0013
	(0.0034)	(0.0013)	(0.0033)	(0.0009)
$\:employ\_type$	0.0099***	−0.00024	0.01***	0.0016)
	(0.0021)	(0.00068)	(0.002)	(0.00082)
R-squared	0.15	0.0095	0.2270	0.1186
Chi-square [prob.]	1560.64[0.000]	44.14[0.0000]	955.40[0.0000]	123.73[0.0000]

Standard errors in parentheses

Source: Authors’ Computation from STATA 15.0

*** *p* < 0.01, ** *p* < 0.05, * *p* < 0.1

**Table 6:** Probit regression results (Average marginal Effects)

VARIABLES	$\:insured$	$\:insure\_cbi$	$\:insure\_ebi$	$\:insure\_pri$
$\:educ\_level$	0.016***	−0.002***	0.021***	0.0024***
	(0.0019)	(0.00067)	(0.0021)	(0.0007)
$\:age\_hh$	0.00014**	0.00004*	0.0001***	0.00002
	(0.000055)	(0.000021)	(0.00004)	(0.00001)
$\:own\_bank\_acc$	0.031***	0.0033***	0.0234***	0.0023***
	(0.0016)	0.00077)	(0.0013)	(0.00048)
$\:wealth$	0.013***	0.000532	0.025***	0.004***
	(0.0021)	(0.0007)	(0.0033)	(0.0013)
$\:marital$	0.0202***	0.00224*	0.019***	0.0012
	(0.00305)	(0.0013)	(0.0028)	(0.0008)
$\:employ\_type$	0.0088***	−0.00024	0.0095***	0.0016**
	(0.0019)	(0.00067)	(0.0018)	(0.0007)
R-squared	0.15	0.0099	0.2286	0.1194
Chi-square [prob.]	1548.40[0.000]	41.83[0.0000]	1020.98[0.0000]	142.83[0.0000]

Standard errors in parentheses

Source: Authors’ Computation from STATA 15.0

*** *p* < 0.01, ** *p* < 0.05, * *p* < 0.1

First, there is a positive relationship between educational level and the likelihood of enrolling for health insurance. The average marginal effects (AME) analysis for the logit models shows that the average probability of enrolment among individuals with high education (secondary and post-secondary education) is higher than that associated with low education (primary and no education) by 1.9, 2.6, and 0.27% points for overall (all types), employer-sponsored, and private insurance, respectively. The results are statistically significant at the 1% level. However, there is an inverse relationship between educational level and enrolment in the case of community-based insurance (see Table 5). The AME results for the probit regression models align with these findings. The average probability of enrolment is higher among individuals with higher levels of education than among those with low education by 1.6, 2.1, and 0.24% points for overall insurance (all types of insurance), employer-sponsored insurance, and private insurance, respectively. Conversely, a negative relation exists between education level and enrolment into CBHI (see Table 6).

Second, there is a positive relationship between the age of the household head and the likelihood of enrolling for health insurance. The AME results for logit indicate that a one-year increase in the age of the household head increases the average probability of enrolment by 0.01% points for overall and employer-sponsored insurance, with the coefficients being statistically significant at the 1% level (see Table 5). The results are not statistically significant for community-based and private insurance at the conventional levels. Similar results are observed from the AME results for the probit model (see Table 6). Third, there is a positive relationship between financial inclusion and the probability of having health insurance. The AME results are consistent across both the logit and probit models. The results from the logit model show that the average probability of insurance uptake, in general, is higher among bank account owners than that associated with non-bank account owners by 3.1, 0.32, 2.5, and 0.23% points for overall, community-based, employer-sponsored, and private insurance, respectively (see Table 5). These regression coefficients are significant at the 1% level. Similar results were obtained for the probit model (see Table 6).

Fourth, there is a positive relationship between wealth level and the probability of having health insurance. The results of the AME analysis for logit models show that the average probability of health insurance uptake among middle- and high-income categories increases by 1.7, 3.1, and 0.51% points, respectively, compared to the low-income categories for overall, employer-sponsored, and private insurance (see Table 5). These regression coefficients are statistically significant at the 1% level. These results are consistent with probit regression models (see Table 6). While low-income groups have a low probability of enrolling in health insurance due to financial constraints, middle- and high-income groups do not face such constraints and are more likely to enroll in health insurance. Fifth, being married increases the chances of an individual enrolling for health insurance. The AME results for the logit model show that the average probability of insurance uptake among married individuals and that associated with unmarried persons by 2.1, 0.2, and 1.9% points for overall, community-based, and employer-sponsored insurance, respectively (see Table 5). Except for community-based and private health insurance, the regression coefficients are statistically significant at the 1% level. However, for the probit model, all coefficients are statistically significant, except for the results related to private health insurance (see Table 6).

Lastly, there is a positive relationship between employment and the probability of having health insurance. The AME results for the logit model indicate that the probability of enrolment among year-round employees is higher than that among seasonal/occasional employees by 0.99, 1.0, and 0.16% points for overall, employer-sponsored, and private insurance, respectively (see Table 5). This suggests that the quality of jobs matters for enrolment in health insurance. The regression coefficients are statistically significant at the 1% level, except for the results for community-based and private insurance. This suggests that the quality of jobs matters for enrolment in health insurance, particularly for employer-sponsored health insurance, because not all jobs offer employees adequate enrolment options. Similar results were obtained for the probit regression models (see Table 6).

## Discussion of results

The regression results presented in Tables 5 and 6 offer important insights into the determinants of health insurance uptake in Nigeria, with implications for the design and expansion of coverage under the National Health Insurance Authority (NHIA) Act.

### Education and health insurance uptake

Education emerges as one of the strongest predictors of health insurance enrolment in Nigeria. The positive effect of high education (secondary and post-secondary education) on overall, employer-sponsored, and private insurance uptake reflects the role of human capital in shaping health-seeking behaviour. Educated individuals are more likely to understand the benefits of prepayment and risk pooling, navigate enrolment processes, and overcome information barriers [see 49, 14]. However, the inverse relationship between education and enrolment in community-based health insurance (CBHI) schemes reflects the perception of CBHI as a mechanism primarily for rural, informal, and low-income populations who are typically excluded from formal employment–linked coverage. This aligns with evidence from other Sub-Saharan African contexts where the better-educated tend to opt for private or employer-sponsored schemes rather than CBHI [31, 32]. In Nigeria, this dichotomy underscores the stratification of enrolment along socioeconomic lines, highlighting the risk that CBHI may remain residual in a comprehensive universal health coverage (UHC) strategy.

### Age and household headship

The positive effect of the household head’s age on enrolment in health insurance is consistent with expectations. Older individuals typically have greater health needs and risk perceptions, making them more willing to invest in insurance protection [50, 51, 52]. In Nigeria, where out-of-pocket (OOP) expenditure still accounts for over 70% of total health spending [3], older household heads may seek insurance as a strategy to mitigate catastrophic health costs for dependents. However, the limited effect of age on CBHI and private insurance uptake suggests that age-driven demand is mediated by scheme design: CBHI is more often promoted in rural communities with younger, agrarian populations, while private schemes cater to wealthier urban groups less constrained by age-specific household dynamics.

### Financial inclusion

The positive and significant relationship between bank account ownership and insurance uptake demonstrates the role of financial inclusion in facilitating health system access. In Nigeria, where nearly 60% of adults remain outside the formal financial system [53], limited access to banking services constrains premium collection and enrolment. The strong relationship between financial inclusion and employer-sponsored or private health insurance uptake suggests that schemes are structured around payroll deductions and electronic payments. This finding reinforces the argument by [54] that digital technologies, including mobile banking, are central to improving insurance penetration. For Nigeria, expanding mobile money and agency banking could significantly improve coverage, especially in rural communities.

### Wealth and insurance enrolment

The results confirm that insurance enrolment in Nigeria remains pro-rich, with middle- and high-income groups significantly more likely to be insured. This finding is in line with studies showing persistent wealth-driven inequities in health coverage [29, 55]. For Nigeria, the heavy reliance on OOP payments has entrenched inequalities, as low-income households often forgo enrolment due to competing subsistence priorities. The implication is that without targeted subsidies, premium waivers, or cross-subsidisation mechanisms, health insurance risks reinforce rather than reduce socioeconomic inequalities. The National Health Insurance Authority Act 2022 makes provisions for a Vulnerable Group Fund, which, if operationalised effectively, could help bridge this gap by financing the enrolment of the poor and vulnerable groups.

### Marriage and household composition

Married individuals are significantly more likely to be insured, particularly under employer-sponsored schemes. This is consistent with evidence that family responsibilities increase the perceived need for financial protection against health shocks [55, 56]. In Nigeria, marriage often extends financial and caregiving obligations to children and extended relatives, making insurance more attractive as a tool for household risk management. Interestingly, the weak effect of marriage on CBHI enrolment suggests that marital status does not necessarily translate into demand for community-based schemes, again pointing to the segmented nature of insurance preferences.

### Employment and job quality

Employment status is also a critical determinant, with year-round employees significantly more likely to be enrolled in health insurance compared to seasonal or occasional workers. This highlights the importance of job quality in determining access to employer-linked insurance [14, 31]. In Nigeria, where informal employment accounts for over 90% of the work-force [10], the dominance of precarious and unregulated jobs limits the effectiveness of employment-based insurance as a pathway to UHC. This finding reinforces calls for CBHI expansion and government-subsidised schemes to target the informal sector, which otherwise risks exclusion from coverage.

### Broader implications

Taken together, these findings suggest that health insurance uptake in Nigeria is strongly patterned by socioeconomic stratification—education, wealth, employment type, and financial inclusion—while demographic factors such as age and marital status play important but secondary roles. The Nigerian context reveals a dual challenge: while formal and private schemes primarily benefit the educated, wealthy, and formally employed, CBHI remains underutilised by precisely the groups it aims to serve. Without stronger integration, subsidies, and digital innovations to improve enrolment, the path to UHC will remain fragmented.

## Conclusion and policy recommendations

The National Health Insurance Authority (NHIA) Act, enacted in 2022, made health insurance mandatory for all citizens and legal residents in Nigeria. Unlike the earlier NHIS, the NHIA aims to expand coverage beyond a narrow group of formal sector workers, boosting enrolment in community-based, private, or public insurance plans operated by both federal and state governments. However, compulsion alone may not guarantee wider uptake, making it critical to understand the socioeconomic factors influencing enrolment, which is the main thrust of this paper. We employed logit and probit models for robustness.

Within the Andersen Behavioural Model framework, this study found that enrolment in health insurance is shaped by both predisposing and enabling factors. For overall insurance (all types of insurance) and employer-sponsored insurance, the six statistically significant determinants include education, age of the household head, bank account ownership, wealth, marital status, and employment type. In community-based schemes, education, bank account ownership, and marital status mattered, with those having low education more likely to enroll, suggesting such schemes favour low-income and informal workers. For private insurance, all factors except age and marital status were statistically significant, with full-time employment, higher education, and wealth level strongly increasing the likelihood of enrolment.

Based on the findings of this study, several policy recommendations are proposed. *First*, community-based insurance schemes may require targeted outreach to less-educated and low-income groups who might find it challenging to access employer-sponsored and private health insurance. *Second*, insurance providers could focus on younger demographics, emphasising long-term financial benefits. *Third*, expanding digital banking and mobile payment platforms can facilitate easier premium payments and enhance enrolment through improved financial inclusion. *Fourth*, the introduction of subsidies or tiered premium structures could make health insurance more accessible to low-income households. *Fifth*, expanding employer-sponsored schemes to include contract and informal sector workers is essential, particularly given that the informal economy accounts for over 90% of the total employment in Nigeria. As posited by Schneider (2004), trust is crucial in community-based health insurance, which hinges on informal agreements and weak legal structures.

While this study offers valuable insights, several limitations should be acknowledged. *First*, the 2018 DHS data is outdated. Prospective researchers should rely on the updated database as soon as it becomes available. *Second*, there is significant value in conducting longitudinal analyses using DHS data across multiple years. Additionally, the socioeconomic factors used in this study are not exhaustive. Hence, prospective researchers could explore other socioeconomic indicators that were not captured in this study, particularly in line with the Andersen Behavioural Model. Finally, extending this research to include DHS data from multiple countries would enable meaningful cross-country comparisons, offering a richer perspective on the subject matter.

## Supplementary Information

Supplementary Material 1.

Supplementary Material 2.

## Data Availability

The authors relied entirely on the Nigerian Demographic and Health Survey data, which can be obtained via: [https://dhsprogram.com/data/available-datasets.cfm](https:/dhsprogram.com/data/available-datasets.cfm). However, the authors can make the specific data on all the variables employed in the study available on request.

## Footnotes

1 Social security was not considered in the regression analysis due to the small number of respondents associated with it.
