# Supplementary material for: Analysing the determinants of healthcare insurance uptake in Nigeria
Source: BMC Health Serv Res. 2025 Oct 6;25:1310. doi: 10.1186/s12913-025-13422-0 (PMC12502249; doi:10.1186/s12913-025-13422-0)
Supplement: Supplementary file 1 — Supplementary Material 1. [file 12913_2025_13422_MOESM1_ESM.pdf]

Supplementary Material

Table I: Logit Regression Results (Log of Odds Estimates)

VARIABLES	*insured*	*insure_cbi*	*insure_ebi*	*insure_pri*
educ_level	0.916***	-0.46***	1.84***	1.238***
	(0.104)	(0.156)	(0.199)	(0.369)
age_hh	0.00688***	0.009*	0.008***	0.0063
	(0.00262)	(0.00492)	(0.0025)	(0.0059)
own_bank_acc	1.530***	0.782***	1.73***	1.089***
	(0.0766)	(0.174)	(0.099)	(0.212)
wealth	0.805***	0.126	2.179***	2.39***
	(0.124)	(0.167)	(0.348))	(0.785)
marital	1.032***	0.536	1.383***	0.585
	(0.164)	(0.328)	(0.228)	(0.418)
employ_type	0.481***	-0.059	0.696***	0.747**
	(0.102)	(0.165)	(0.142)	(0.378)
constant	-7.299***	-6.390***	-10.569***	-10.976***
	(0.238)	(0.427)	(0.425)	(0.759)

Standard errors in parentheses
*** p<0.01, ** p<0.05, * p<0.1

Note: The logit models report logit coefficients, which represent the change in the log of odds of being insured for a one-unit increase in each predictor. All reported values are logit coefficients, meaning they represent the change in the log of odds of being insured for a one-unit increase in each independent variable.

Source: Authors' Computation from STATA 15.0

Table II: Probit Regression Results (Z-score Estimates)

VARIABLES	*insured*	*insure_cbi*	*insure_ebi*	*insure_pri*
educ_level	0.344***	-0.165***	0.671***	0.3893***
	(0.0401)	(0.055)	(0.0656)	(0.1089)
age_hh	0.00303**	0.003*	0.0038***	0.0027
	(0.00120)	(0.0017)	(0.0012)	(0.0021)
own_bank_acc	0.684***	0.277***	0.747***	0.3699***
	(0.0327)	(0.0617)	(0.0384)	(0.070)
wealth	0.286***	0.044	0.789***	0.684***
	(0.0455)	(0.0569)	(0.1033)	(0.2011)
marital	0.443***	0.186**	0.592***	0.2004
	(0.0661)	(0.105)	(0.0883)	(0.135)
employ_type	0.192***	-0.0201	0.303***	0.2675**
	(0.0414)	(0.0554)	(0.0579)	(0.116)
constant	-3.398***	-2.958***	-4.6519	-4.376***
	(0.0963)	(0.1417)	(0.1581)	(0.211)

Standard errors in parentheses
*** p<0.01, ** p<0.05, * p<0.1

Note: The probit models report probit coefficients, which represent the change in the z-score (standard normal distribution) of being insured for a one-unit increase in each predictor.

Source: Authors' Computation from STATA 15.0
